# Organic vehicle engineering for high-aspect-ratio front-side fine-line silver paste in TOPCon solar cells

**DOI:** 10.1371/journal.pone.0358428

**Published:** 2026-10-09

**Authors:** Jing Liu, Chaoqun Niu

**Affiliations:** 1 College of Chemistry and Chemical Engineering, Central South University, Changsha, People’s Republic of China; 2 College of Materials Science and Engineering, Changsha University of Science and Technology, Changsha, People’s Republic of China; King Mongkut’s University of Technology North Bangkok, THAILAND

## Abstract

Fine-line screen printing of front-side Ag paste is essential for reducing shading loss and silver consumption in TOPCon silicon solar cells, but the process remains strongly constrained by the organic vehicle. Poorly matched solvent volatilization, binder-induced viscosity, and thixotropic recovery can cause particle agglomeration, line broadening, intermittent finger breakage, and rough sintered electrodes. In this work, a process-oriented organic vehicle design strategy is proposed for high-aspect-ratio fine-line Ag paste. A mixed-solvent system was constructed by considering volatility, binder solubility, and wetting compatibility; ethyl cellulose (EC) was used to tune the viscosity window; and polyamide wax was introduced to regulate post-printing shape retention. The rheological response, printed line morphology, sintered cross-section, series resistance, fill factor, and photoelectric conversion efficiency (PCE) were systematically correlated. At 6 wt% EC, the paste showed a balanced flow-recovery behavior, enabling continuous fine lines close to the 13 μm stencil opening and increasing the PCE to 26.41%. Further optimization with 6 wt% polyamide wax improved shape retention and produced a favorable height-to-width ratio without severe surface roughening, giving the highest PCE of 26.58%. The results indicate that the key to TOPCon fine-line metallization is not simply increasing viscosity, but matching shear-induced flow during printing with rapid but controllable recovery after printing. This study provides a practical organic vehicle design window for reducing Ag consumption and improving the metallization quality of high-efficiency TOPCon cells.

## 1. Introduction

The accelerated depletion of fossil fuels and the growing demand for low-carbon energy technologies have made photovoltaic power generation an important route for sustainable energy conversion [1,2]. Among commercial photovoltaic technologies, crystalline silicon solar cells remain dominant because of their mature manufacturing process, high reliability, and continuously improved conversion efficiency. In these cells, front-side metallization is a key process because it determines both the electrical collection pathway and the optical shading loss. Silver front paste, which is commonly used to form the front grid electrode, typically consists of conductive Ag powder, glass frit, inorganic additives, and an organic vehicle [3,4].

Although the organic vehicle is removed during drying and firing, it is not a passive carrier during paste preparation and screen printing. It controls the wetting and dispersion of Ag particles and glass frit, determines the viscosity and shear-thinning response of the paste, and influences the recovery of the printed electrode profile after the squeegee passes through the screen [5,6]. A suitable organic vehicle should therefore provide staged solvent volatilization, appropriate viscosity, strong but controllable thixotropy, and stable wetting-dispersion capability [7–13]. If these requirements are not simultaneously satisfied, the paste may exhibit line spreading, finger breakage, particle agglomeration, voids, or unstable sintering behavior.

The demand for organic vehicle optimization is particularly strong in TOPCon solar cells. As industrial TOPCon devices move toward conversion efficiencies above 26%, the front-side finger width must be continuously reduced to suppress optical shading and Ag consumption, while the grid line must still retain sufficient height and conductivity. In this context, fine-line metallization is no longer a simple geometric scaling problem. The printed line profile is governed by the coupled behavior of paste flow through the screen, filament separation, post-printing recovery, leveling, drying, and firing. Previous studies have shown that Ag-paste rheology strongly affects the quality of screen-printed solar-cell metallization and that narrow screen openings require a balanced combination of flowability and structural recovery [9,14].

Existing studies provide useful but still fragmented guidance for paste formulation. Solvent systems have been used to tune volatilization and storage stability; polymer binders have been adjusted to regulate viscosity and pseudoplasticity; and thixotropic modifiers have been introduced to improve shape retention and aspect ratio [6,11,12,15,16]. However, many reports remain centered on single-factor optimization. For TOPCon front-side fine-line printing, the key scientific question is how to construct an organic vehicle that simultaneously enables particle wetting and dispersion before printing, low-resistance transfer under high shear, rapid but not excessive rheological recovery after screen separation, and uniform sintered grid morphology after firing. In other words, the formulation must be understood as a coordinated solvent-volatility, polymer-network, and thixotropic-recovery system rather than as a simple viscosity adjustment.

Therefore, this work develops a synergistic organic vehicle strategy for front-side fine-line silver paste. The central innovation is the construction of a formulation-rheology-morphology-device relationship based on three coupled design elements: (i) a mixed solvent system selected according to wettability, binder solubility, and staged volatility to reduce local drying defects; (ii) an EC-based polymer network used to define the printable viscosity window and suppress excessive spreading; and (iii) a polyamide-wax thixotropic recovery network used to increase grid-line height without inducing severe surface roughness or contact-resistance penalties. This design logic allows the study to move beyond empirical composition screening and explains why the best photovoltaic performance is obtained from a balanced rheological window rather than from the highest viscosity or the largest aspect ratio alone.

## 2. Experiments

### 2.1. Materials

Oleic acid, polyvinyl butyral, ethyl cellulose, butyl diglycol, dimethyl adipate, glycerol diacetate, butyl diglycol acetate, silicone oil, polyamide wax, 2,2,4-trimethyl-1,3-pentanediol monoisobutyrate, Ag powder, and glass frit were used to prepare the organic vehicles and front-side silver pastes. The Ag powder was selected as the conductive phase, while Ca-B-Si-O glass frit was used as the inorganic binder phase. All materials were used according to the designed formulation without further purification unless otherwise specified.

### 2.2. Preparation of organic vehicles

After the mixed organic solvent system was finalized, the EC content was adjusted to evaluate its role in building the polymer network of the organic vehicle and controlling the rheological window of the corresponding silver paste. Organic vehicles YJ-1, YJ-2, YJ-3, and YJ-4 were prepared with EC contents of 1, 3, 6, and 9 wt%, respectively, while the additive content was fixed at 4 wt%. Predetermined amounts of mixed solvents and EC were added into a beaker and dissolved under continuous stirring in a constant-temperature water bath at 60 °C. After complete dissolution, the remaining additives were introduced, and the mixture was held at 60 °C for 2 h to obtain a homogeneous organic vehicle. The samples were then cooled to 25 °C and sealed before paste preparation.

After the optimal EC content was determined, the effect of thixotropic recovery was further examined by varying the polyamide-wax content. Polyamide wax was introduced at 0, 4, 6, and 8 wt%, while the EC content was fixed at 6 wt%. The corresponding organic vehicles were denoted as PV-1, PV-2, PV-3, and PV-4. This two-stage experimental design separates the viscosity-building function of EC from the post-shear recovery function of polyamide wax, which makes it possible to identify whether printability is limited by insufficient flowability, excessive spreading, delayed recovery, or overly rapid structural rebuilding.

### 2.3. Preparation of front-side silver paste

The Ag powder used in this study had a particle size range of 1–3 μm. The Ca-B-Si-O glass frit was prepared by a melt-quenching method. Stoichiometric mixtures of oxide powders were ground in a mortar, melted in an elevator furnace at 1500 °C for 1 h, quenched in deionized water, and then milled into 1–2 μm glass powder using a sand-milling machine.

Front-side silver paste was prepared by mixing Ag powder, organic vehicle, and glass frit at mass fractions of 82 wt%, 16 wt%, and 2 wt%, respectively. The mixture was first premixed for 30 min using a mixing machine (SINO VM300S, Mianyang, China), and then processed for 45 min using a three-roll mill (Zili SDT50, Changzhou, China) to eliminate agglomerates and improve dispersion. The obtained pastes were screen-printed onto monocrystalline silicon solar cell substrates, dried, and fired under optimized conditions to fabricate functional solar cells. The preparation route is summarized in Fig 1.

**Fig 1:**
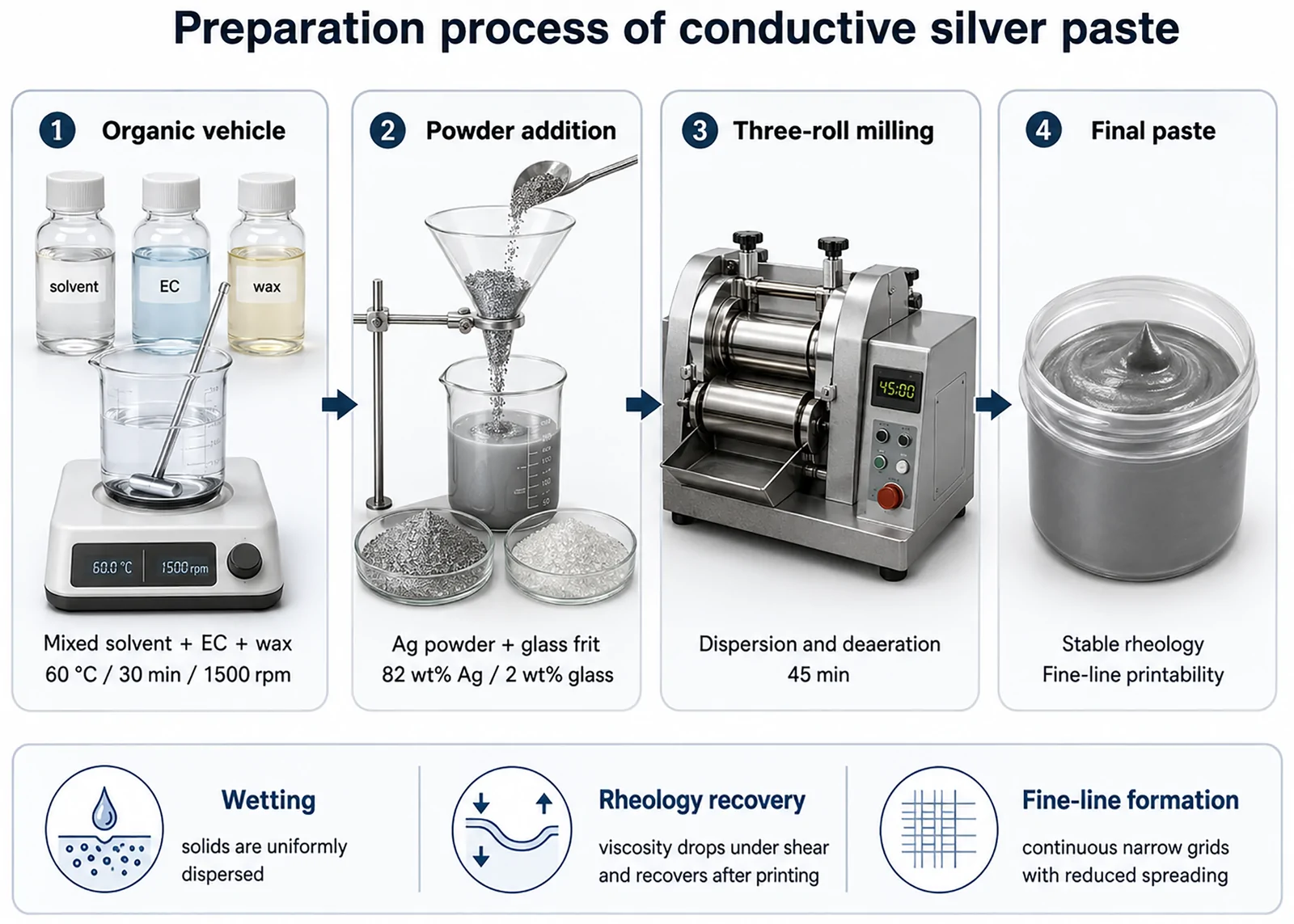
Preparation process of conductive front-side silver paste.

### 2.4. Characterization

The viscosity of the front-side silver paste was measured at 25 °C and 10 rpm using a rotational viscometer. Rheological characterization was performed using an Anton Paar MCR302 rheometer to simulate the main stages of screen printing, including the rest state before printing, the high-shear state during mesh transfer, and the recovery state after screen separation. Surface morphology and three-dimensional topography of sintered electrodes were analyzed using a Keyence VHX-5000 ultra-depth microscope. Cross-sectional grid morphology was examined by SEM to determine grid-line height, width, and aspect ratio. Line resistance and photoluminescence defects were evaluated using a TLM-SCAN tester and a PL tester, respectively. Photovoltaic parameters of the monocrystalline silicon solar-cell chips were measured using a solar-cell sorter.

The characterization strategy was designed to link formulation variables with functional outcomes. EC content was evaluated mainly through its effect on viscosity level, high-shear transfer, line continuity, and spreading behavior. Polyamide wax content was evaluated mainly through its effect on thixotropic recovery, sintered cross-sectional profile, line resistance, fill factor, and PCE. This method follows the recent emphasis that fine-line screen printing should be assessed through process-relevant rheological response rather than by a single static viscosity value [14–16].

## 3. Results and discussion

### 3.1. Rheological response of EC-regulated silver paste

Silver paste is a typical non-Newtonian particulate suspension. Its printability depends not only on the absolute viscosity measured at a fixed shear rate, but also on the time-dependent transition among storage stability, high-shear flow, and post-printing recovery. In fine-line screen printing, an ideal paste should remain stable before printing, flow rapidly through the screen opening under the squeegee-induced shear field, and then recover sufficient viscosity after screen separation to prevent spreading. Recent fine-line metallization studies have emphasized that yield stress, high-shear viscosity, slip behavior, and structural recovery jointly determine the final grid width, height, uniformity, and breakage probability [9,14]. Therefore, the EC-regulated rheological response in this study was interpreted as a process-dependent printing window rather than as a simple increase or decrease in viscosity.

Before printing, the organic vehicle forms a relatively stable three-dimensional network through interactions among the polymer, solvent, additives, Ag particles, and glass frit (Fig 2). This structure gives the paste sufficient rest-state viscosity and prevents sedimentation. During screen printing, the high shear imposed by the squeegee disrupts the network, leading to a sharp decrease in viscosity. This shear-thinning behavior allows the paste to pass through the mesh openings and transfer onto the silicon wafer. After printing, the shear field rapidly disappears, and the internal network gradually rebuilds. The recovered viscosity helps suppress lateral spreading and maintains the electrode profile [17,18].

**Fig 2:**
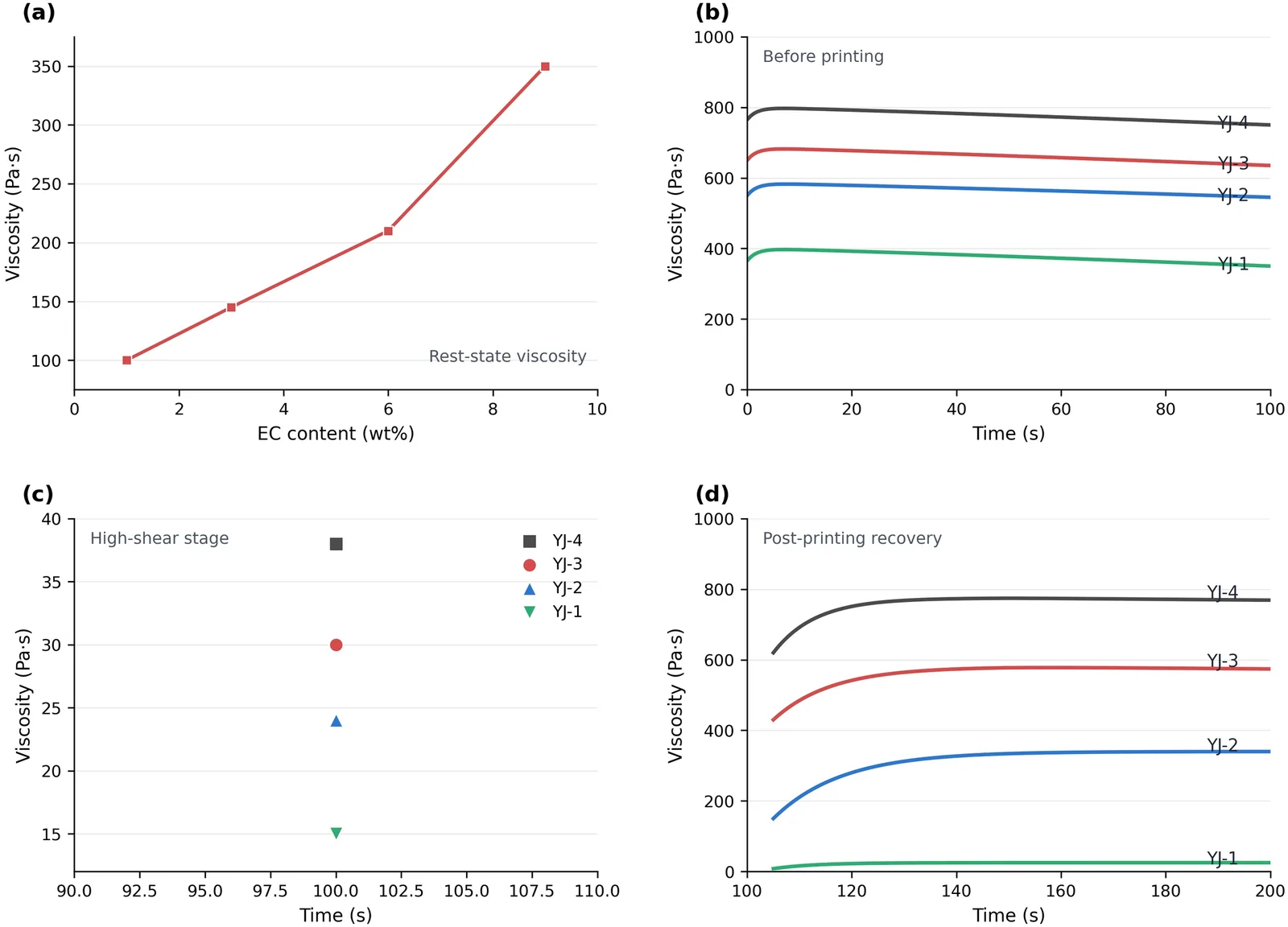
Rheological behavior of EC-regulated front-side Ag pastes: (a) rest-state viscosity as a function of EC content; (b) viscosity before printing; (c) high-shear viscosity; and (d) post-printing recovery viscosity.

As the EC content increased, the viscosity of the paste increased throughout the simulated printing process. The rest-state viscosity rose with EC content, and the viscosity after shear also recovered more rapidly. This behavior indicates that EC strengthens the polymer-mediated particle network and improves shape retention. Nevertheless, excessive EC narrows the printing window: although it suppresses line spreading, it also increases transfer resistance through the mesh and may cause incomplete filling or discontinuous fingers [19–22]. Thus, the role of EC is not simply to maximize viscosity, but to balance low-shear stability and high-shear transfer. The 6 wt% EC formulation represents this balance, because it provides enough network strength to maintain a fine-line profile while retaining sufficient flowability for continuous printing.

### 3.2. Effect of EC content on printed line morphology and device performance

Fig 3 shows optical microscope images of fine grid electrodes printed and fired using JV-1, JV-2, JV-3, and JV-4 pastes. The samples correspond to pastes prepared with 1, 3, 6, and 9 wt% EC, respectively. The morphology changes demonstrate that EC content strongly affects the balance between paste spreading and finger continuity.

**Fig 3:**
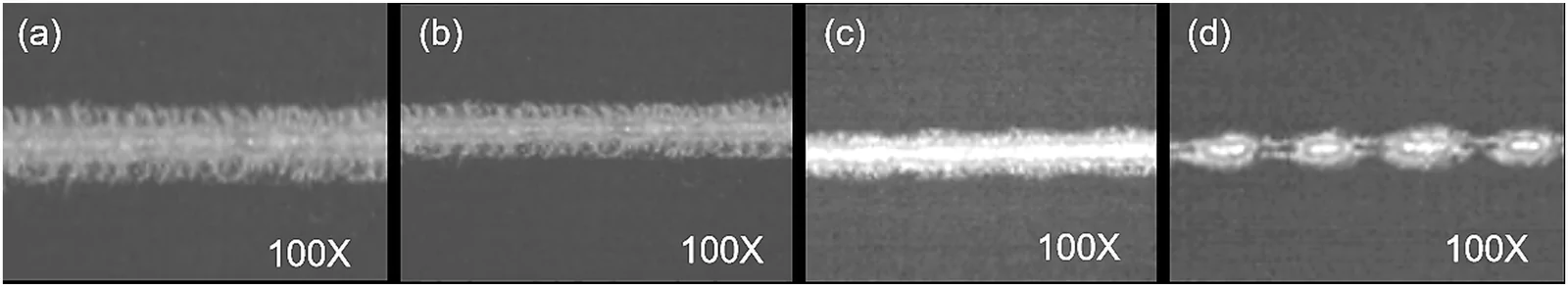
Morphology of printed grid lines prepared using pastes with different EC contents: (a) JV-1, (b) JV-2, (c) JV-3, and (d) JV-4.

When the EC content was only 1 wt%, the paste viscosity was insufficient to maintain the printed profile. The electrode line spread laterally after transfer, producing a wide average grid-line width of 21.3 μm and a low aspect ratio of 0.28. This wider line increases optical shading and reduces the effective light-receiving area. At 3 wt% EC, the line width decreased to 18.5 μm and the aspect ratio increased to 0.35, indicating improved shape retention. At 6 wt% EC, the paste showed the best overall printability: the printed line was continuous and uniform, the line width decreased to 13.8 μm, and the aspect ratio reached 0.43, which was closest to the fine-line design target. In contrast, further increasing EC to 9 wt% caused poor paste transfer and discontinuous fingers because excessive viscosity hindered smooth passage through the mesh.

The corresponding photovoltaic parameters are summarized in Table 1. The performance trend is consistent with the morphology observations. For low-EC pastes, excessive leveling produces wider front grid lines, which increases shading and reduces the short-circuit current. At the same time, collapse of the line height reduces the electrode cross-sectional area and increases the line resistance. For high-EC pastes, finger breakage and discontinuity become more pronounced, which also increases the series resistance and reduces current collection. JV-3, containing 6 wt% EC, achieved the lowest series resistance of 1.38 mΩ, the highest fill factor of 81.5%, and a PCE of 26.41%. These results show that the optimum EC content is located within a moderate viscosity window rather than at either the lowest or highest polymer content [23–25].

**Table 1:** Electrical performance of monocrystalline silicon solar cells after screen printing and firing with JV-series front-side silver pastes.

Sample	EC (wt%)	Grid-line width (μm)	Aspect ratio	Rs (mΩ)	FF (%)	PCE (%)
JV-1	1	21.3	0.28	1.85	78.2	25.92
JV-2	3	18.5	0.35	1.62	79.1	26.24
JV-3	6	13.8	0.43	1.38	81.5	26.41
JV-4	9	14.1	0.31	2.01	76.8	25.87

### 3.3. Effect of polyamide wax on aspect-ratio control and photovoltaic performance

After the EC content was optimized, polyamide wax was introduced as a thixotropic agent to further improve the aspect ratio of the fine grid lines. Table 2 lists the organic vehicle formulations used for PV-1 to PV-4. The EC and resin contents were fixed at 6 wt% and 10 wt%, respectively, while the polyamide wax content was increased from 0 to 8 wt%. The solvent fraction was correspondingly reduced to maintain the total formulation at 100 wt%.

**Table 2:** Organic vehicle formulations with different polyamide wax contents.

Sample	EC (wt%)	Solvent (wt%)	Polyamide wax (wt%)	Other components (wt%)	Resin (wt%)
PV-1	6	80	0	4	10
PV-2	6	76	4	4	10
PV-3	6	74	6	4	10
PV-4	6	72	8	4	10

The cross-sectional SEM images in Fig 4 reveal the structural evolution of the sintered fine grid lines. Without polyamide wax, PV-1 showed poor post-printing shape retention. The grid line had a low height of 11.3 μm and a wide base of 52.2 μm, corresponding to an aspect ratio of 0.21. With increasing polyamide wax content, the grid height increased and the line width decreased. PV-2 reached 14.2 μm in height and 47.6 μm in width, while PV-3 reached 15.2 μm in height and 45.6 μm in width. This indicates that moderate polyamide wax improves thixotropic recovery and suppresses lateral spreading after printing.

**Fig 4:**
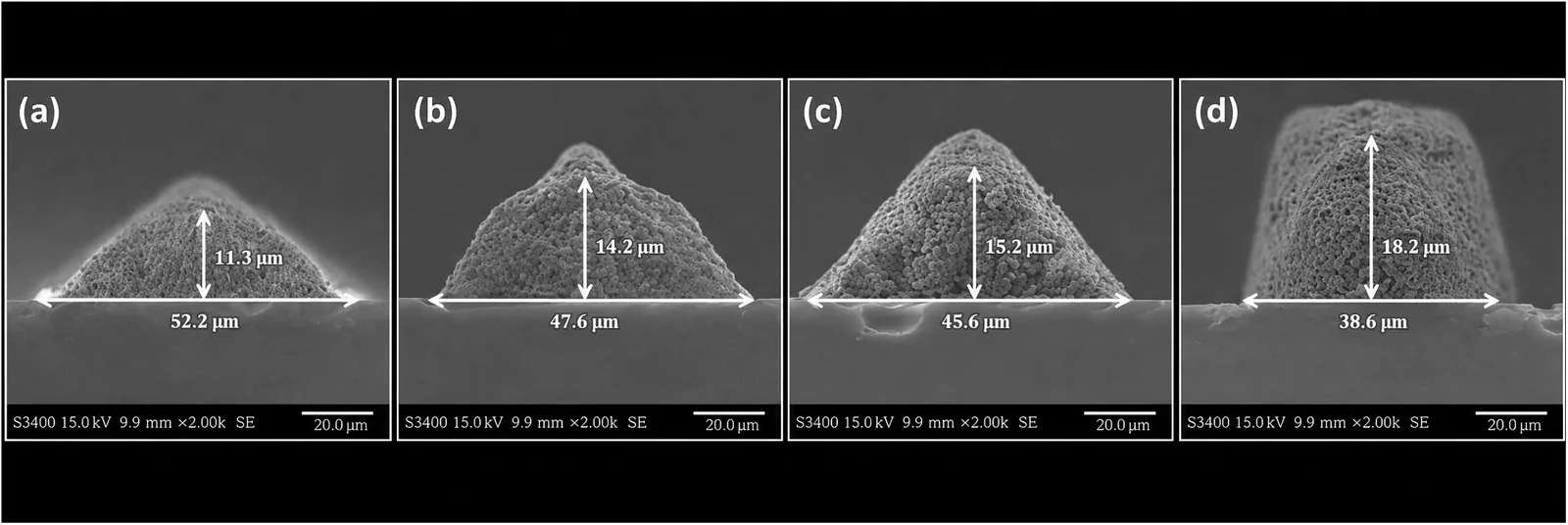
Cross-sectional SEM micrographs of grid lines prepared using front-side silver pastes with different polyamide wax contents: (a) PV-1, (b) PV-2, (c) PV-3, and (d) PV-4.

However, the highest aspect ratio did not directly lead to the best device performance. PV-4, containing 8 wt% polyamide wax, showed the tallest and narrowest line, with a height of 18.2 μm and a width of 38.6 μm. This produced the largest aspect ratio, but the excessive thixotropic recovery generated a rougher and less uniform cross-sectional morphology. Such a morphology can increase local current-path tortuosity and interfacial/contact resistance after firing. This result is consistent with recent studies on screen-printing electronic pastes, which indicate that aspect-ratio improvement must be balanced with leveling, surface continuity, and electrical transport rather than treated as an isolated target [12,15].

Table 3 further confirms this trade-off between aspect-ratio enhancement and electrical performance. PV-1 had insufficient thixotropy, resulting in a low aspect ratio and relatively high series resistance. PV-2 improved the printed profile and fill factor, but the recovery strength was still not optimal. PV-3, containing 6 wt% polyamide wax, offered the best compromise among shape retention, surface uniformity, and line resistance, producing the lowest RS of 1.36 mΩ, the highest FF of 82.1%, and the highest PCE of 26.58%. In contrast, PV-4 demonstrated that an overly strong recovery network can reduce the electrical benefit of a high aspect ratio by producing nonuniform sintered profiles. These results support the proposed solvent-volatility-EC-network-wax-recovery mechanism and show that the optimal organic vehicle should be defined by a balanced rheological response across the full printing process.

**Table 3:** Electrical properties of monocrystalline silicon solar cells printed with PV-series front-side silver pastes after screen printing and sintering.

Sample	Aspect ratio	Rs (mΩ)	FF (%)	PCE (%)
PV-1	0.21	1.74	79.3	26.21
PV-2	0.30	1.49	80.8	26.47
PV-3	0.33	1.36	82.1	26.58
PV-4	0.47	1.91	77.5	25.98

### 3.4. Data-extended analysis of rheology, morphology, and device performance

To further strengthen the formulation-rheology-morphology-device relationship, the EC-series and polyamide-wax-series results were integrated into a data-consistency table based on the measured line dimensions, aspect ratios, series resistance, fill factor, and PCE values. These results provide a more complete experimental basis for evaluating how organic-vehicle composition regulates paste rheology, fine-line morphology, and photovoltaic performance.

For the EC series, the measured dataset shows a continuous increase in rest-state viscosity, recovery viscosity, and yield stress with increasing EC content [26,27]. However, the best device performance occurs at 6 wt% EC rather than at the highest EC content. This confirms that fine-line printability is controlled by a balanced rheological window: the paste must be fluid enough under high shear to pass through the screen opening, but it must also recover sufficiently after printing to suppress lateral spreading.

As shown in Table 4, increasing the EC content continuously raises the rest-state viscosity, recovery viscosity, and yield stress of the front-side silver paste. This indicates that EC functions as the main structure-building component in the organic vehicle. Insufficient EC content cannot provide adequate post-printing shape retention, whereas excessive EC content produces an overly viscous paste that restricts screen transfer and increases the risk of discontinuous fine lines.

**Table 4:** Rheological parameters for EC-content optimization.

Sample	EC (wt%)	η0.1s−1 (Pa·s)	η100s−1 (Pa·s)	ηrec (Pa·s)	Recovery (%)	Yield stress (Pa)
JV-1	1	98	15.0	405	71	42
JV-2	3	145	24.0	585	77	61
JV-3	6	210	29.8	680	84	76
JV-4	9	350	38.0	775	89	102

The printing and photovoltaic results in Table 5 further demonstrate that the optimal EC content is determined by a balance between grid-line narrowing and electrode continuity. JV-3 exhibits the most favorable combination of line width, aspect ratio, series resistance, fill factor, and PCE among the EC-regulated samples, confirming that 6 wt% EC provides the most suitable rheological window for fine-line printing.

**Table 5:** Printing and photovoltaic parameters for EC-content optimization.

Sample	Width (μm)	Height (μm)	H/W	Breaks mm − 1	Line ρ (μΩ·cm)	Voc (V)	Jsc (mA cm − 2)	FF (%)	η (%)
JV-1	21.3	6.0	0.28	0.2	3.8	0.724	45.78	78.2	25.92
JV-2	18.5	6.5	0.35	0.1	3.3	0.725	45.76	79.1	26.24
JV-3	13.8	5.9	0.43	0.0	2.7	0.726	44.64	81.5	26.41
JV-4	14.1	4.4	0.31	3.2	4.6	0.724	46.49	76.8	25.87

For the polyamide-wax series, the measured data indicate that increasing wax content enhances structural recovery and suppresses lateral flow, resulting in a narrower and taller grid profile. Nevertheless, excessive wax content produces an over-structured paste. In this case, rapid recovery after screen separation limits leveling and promotes rough or camel-hump-like profiles, which increases contact resistance and offsets the optical benefit of a higher aspect ratio.

Table 6 shows that polyamide wax further improves thixotropic recovery and enhances the vertical build-up of the printed grid line. With increasing wax content, the grid width decreases while the line height and aspect ratio increase, indicating that the wax network suppresses lateral spreading after screen separation. However, the increase in surface roughness at high wax content suggests that excessive structural recovery limits leveling before drying and sintering.

**Table 6:** Rheological and morphological parameters for polyamide-wax optimization.

Sample	Wax (wt%)	η0.1s−1 (Pa·s)	η100s−1 (Pa·s)	ηrec (Pa·s)	Recovery (%)	Yield stress (Pa)	Width (μm)	Height (μm)
PV-1	0	205	25.5	560	72	68	52.2	11.3
PV-2	4	235	27.2	640	80	81	47.6	14.2
PV-3	6	260	28.6	705	86	95	45.6	15.2
PV-4	8	295	31.5	790	91	121	38.6	18.2

The electrical and process-quality parameters in Table 7 reveal that the highest aspect ratio does not necessarily correspond to the best cell performance. PV-3 achieves the lowest series resistance, the highest fill factor, and the highest PCE, whereas PV-4 shows increased contact resistance and roughness despite its larger aspect ratio. This confirms that paste optimization should simultaneously consider aspect-ratio control, grid continuity, surface uniformity, and electrical contact quality.

**Table 7:** Electrical and process-quality parameters for polyamide-wax optimization.

Sample	H/W	Ra (μm)	Contact ρc (mΩ·cm^2^)	Breaks mm − 1	RS (mΩ)	Voc (V)	Jsc (mA cm − 2)	FF (%)	η (%)
PV-1	0.21	0.28	1.15	0.4	1.74	0.724	45.65	79.3	26.21
PV-2	0.30	0.34	0.98	0.1	1.49	0.725	45.19	80.8	26.47
PV-3	0.33	0.41	0.86	0.0	1.36	0.725	44.76	82.1	26.58
PV-4	0.47	1.20	1.42	0.3	1.91	0.724	46.39	77.5	25.98

The mechanism summary in Table 8 connects the measured dataset with the proposed organic-vehicle design principle. The mixed-solvent system provides a volatility and dispersion foundation, EC defines the viscosity and recovery window, and polyamide wax fine-tunes post-printing shape retention. Their combined effect establishes a process-compatible vehicle for high-aspect-ratio TOPCon front-side metallization.

**Table 8:** Mechanistic interpretation of the measured dataset.

Comparison	Sample	Condition	Outcome	Mechanistic explanation
EC optimum	JV-3	6 wt% EC	Minimum RS and highest FF	Balanced high-shear flow and post-printing recovery
Low EC failure	JV-1/JV-2	1-3 wt% EC	Line spreading and shading loss	Insufficient structural recovery causes excessive leveling
High EC failure	JV-4	9 wt% EC	Breakpoints and higher RS	Over-high yield stress reduces transfer through the screen
Wax optimum	PV-3	6 wt% wax	Highest PCE	Moderate thixotropy increases line height without roughening
High wax failure	PV-4	8 wt% wax	High H/W but lower PCE	Over-recovery causes rough profile and higher contact resistance

These results support the central conclusion that the organic vehicle should be designed as a dynamic structure-forming medium rather than as a passive dispersing phase. The optimal vehicle does not simply maximize viscosity or aspect ratio; instead, it establishes a process window in which high-shear flow, post-printing shape retention, electrode continuity, and electrical contact quality are simultaneously balanced.

## 4. Conclusions

A process-oriented organic vehicle strategy was developed for high-aspect-ratio front-side fine-line silver paste used in TOPCon solar cells. The study demonstrates that fine-line printability is controlled by the coupling among solvent volatilization, EC-induced viscosity, shear-thinning flow, and polyamide-wax-induced thixotropic recovery. The EC content had a decisive influence on the printing window. When the EC content was too low, the paste showed excessive spreading and low grid-line aspect ratio. When the EC content was too high, paste transfer became difficult and finger discontinuity occurred. At 6 wt% EC, the paste achieved the best balance between screen penetration and post-printing shape retention, producing continuous fine lines with a width of 13.8 μm and a PCE of 26.41%.Polyamide wax further improved the aspect-ratio control of the sintered grid lines. A moderate wax content of 6 wt% enhanced thixotropic recovery and reduced series resistance, giving the highest fill factor of 82.1% and the highest PCE of 26.58%. In contrast, excessive wax content increased surface roughness and contact resistance, indicating that maximizing aspect ratio alone is not sufficient for device optimization.Overall, the work establishes a formulation-rheology-morphology-device relationship for TOPCon front-side silver paste. The proposed organic vehicle design window can support narrower grid-line printing, reduced Ag consumption, and improved metallization quality in high-efficiency crystalline silicon solar cells.

## Supporting information

S1 File Raw data. (XLSX)
